# Pregnancies through oocyte donation. A mini review of pathways involved in placental dysfunction

**DOI:** 10.3389/fmed.2024.1338516

**Published:** 2024-01-17

**Authors:** Javier Caradeux, Benjamín Fernández, Francisco Ávila, Andrés Valenzuela, Mauricio Mondión, Francesc Figueras

**Affiliations:** ^1^Department of Obstetrics and Gynecology, Clínica Santa María, Santiago, Chile; ^2^Shady Groove Fertility, Santiago, Chile; ^3^Fetal Medicine Research Center, BCNatal, Barcelona Center for Maternal-Fetal and Neonatal Medicine (Hospital Clínic and Hospital Sant Joan de Deu), Institut Clínic de Ginecologia, Obstetrícia i Neonatologia, Universitat de Barcelona, Barcelona, Spain

**Keywords:** *in-vitro*, infertility, preeclampsia, perinatal outcome, immune tolerance

## Abstract

Pregnancies resulting from assisted reproductive techniques (ART) are increasingly prevalent worldwide. While most pregnancies conceived through *in-vitro* fertilization (IVF) progress without complications, mounting evidence suggests that these pregnancies are at a heightened risk of adverse perinatal outcomes. Specifically, IVF pregnancies involving oocyte donation have garnered attention due to numerous reports indicating an elevated risk profile for pregnancy-related complications within this subgroup of patients. The precise mechanisms contributing to this increased risk of complications remain incompletely understood. Nonetheless, it is likely that they are mediated by an abnormal immune response at the fetal–maternal interface. Additionally, these outcomes may be influenced by baseline patient characteristics, such as the etiology of infertility, absence of corpus luteum, and variations in endometrial preparation protocols, among other factors. This review aims to succinctly summarize the most widely accepted mechanisms that potentially contribute to the onset of placental dysfunction in pregnancies conceived through oocyte donation.

## Introduction

Pregnancies through assisted reproductive techniques (ART) are on the rise worldwide; current estimates report nearly 3.2 million cycles per year, with Asia, Europe, and North America as the major contributors (1). The increasing numbers can be partially explained by lower cost and easier access to ART facilities (2, 3), a progressive delay in maternal age at first pregnancy (4, 5), and policymaking and social acceptance of non-traditional families (6, 7).

Even though most pregnancies through *in-vitro* fertilization (IVF) evolve without pregnancy-related complications (8, 9), there is growing evidence that these pregnancies are at higher risk of adverse perinatal outcomes such as preterm birth, preeclampsia, fetal growth restriction and stillbirth (10–15). Recently, the Society of Maternal & Fetal Medicine (SMFM) released a series of recommendations highlighting the need for proper study and management (16).

The exact mechanisms that lead to the increased risk of pregnancy complications are not fully understood, they are probably mediated by baseline characteristics such as maternal age and comorbidities, intrinsic factors of infertility and the interventions carried out during the fertilization process. This is reflected in how perinatal risks vary according to the fertilization method used, the endometrial preparation protocols, the presence of corpus luteum, the selected transfer method (frozen vs. fresh embryo transfer), and the origin of the selected oocyte (12, 14, 17–22).

## Donated oocytes, perinatal outcomes, and placental dysfunction

Among ART, IVF pregnancies through oocyte donation (OD) represent roughly 5 to 7% of all embryo transfers (4, 23), also with an increasing trend over time. In the last few years, these pregnancies have gained attention as several reports have demonstrated a higher risk profile of pregnancy-related complications (24–26).

When compared against IVF conceived with autologous oocytes, pregnancies from OD have shown lower placental volumes at the first trimester (27), a different uterine perfusion profile across gestation (28, 29), higher rates of villitis of unknown etiology (VUE) (30) and also an increased risk of preeclampsia (14, 26) and placental related disease in the third trimester (14, 17, 31–33).

More recently a retrospective study conducted by our group (34), compared antenatal indicators of placental dysfunction between donated and autologous oocytes in the third trimester, demonstrating an abnormal growth velocity from the second trimester to delivery among ART gestations, especially in those conceived with donated oocytes. These findings support mechanisms related to progressive placental dysfunction, rather than abnormal placentation.

## Mechanisms involved in placental dysfunction in OD

Placental dysfunction can manifest in different ways, such as preterm birth, fetal growth restriction, preeclampsia and stillbirth, among others (35–38). In line with the above, several mechanisms have been involved in the onset of placental dysfunction and preeclampsia (39) among pregnancies conceived through OD. In the following sections, these pathways are addressed.

### Baseline characteristics and infertility etiology

Among infertile couples, several baseline characteristics could be related to a higher risk of placental dysfunction and preeclampsia. Among them maternal age is still one of the main factors related with IVF success (40, 41). While this is true for IVF with autologous oocytes, some studies have shown that pregnancy outcomes (i.e., cumulative live rate) among gestations conceived through OD depend mainly on donor age (42, 43). However, the former seems to not apply when it comes to the risk of placental related disease (44). In fact, several factors may interact as mediators for placental dysfunction; first, there is consistent evidence that women with advanced maternal age have more comorbidities, a higher risk of preeclampsia and present more complicated patterns of multimorbidity during pregnancy (45–47). Second, endometrial receptivity has been proposed to be negatively affected by age, potentially influencing implantation, placental function and pregnancy outcome (48, 49), yet further studies are needed. Finally, infertility etiology could also influence pregnancy outcomes; A diminished ovarian reserve, which is a common indication of ART with OD, has been proposed as an indicator of a reduced vascular capacity and has been independently associated with a higher risk of preeclampsia and placental malperfusion lesions (50, 51). Also, premature ovarian failure, recurrent pregnancy loss and idiopathic infertility have been related with several underlying autoimmune diseases (i.e., systemic lupus erythematosus and antiphospholipid syndrome) (52–54), all conditions highly related with placental dysfunction, preeclampsia and adverse pregnancy outcome (55, 56). Other conditions such as endometriosis have been related with a reduced oocyte yield and a dysregulated decidualization leading to a reduced fertilization rate and a higher risk of preeclampsia (57–61). Also, polycystic ovarian syndrome has been related with an increased oxidative stress and chronic inflammation leading to a higher risk of VUE and hypertensive disorder of pregnancy (62–67). Moreover, altered pathways in lipids and glucose metabolism have been proposed to lead to altered placental structure, villous overcrowding, and finally abnormal placental function (68–70). Although by themselves they do not constitute a frequent indication for OD, they may coexist and act as contributing factors to placental dysfunction.

### Embryo transfer method, endometrial preparation protocols, and role of corpus luteum

Several publications demonstrate a different risk profile according to the selected ART protocol (71, 72). Overall, most evidence supports that frozen embryo transfer (FET) presents (among others) a lower risk of small for gestational age and perinatal mortality, but a higher-risk of preeclampsia and placental disease when compared with fresh embryo transfer (14, 71, 73). Regardless, in the last few years the use of FET has presented a progressive increase (23), in part due to a reduced risk of ovarian hyperstimulation syndrome and the expansion of the “freeze all” strategy (which facilitates single embryo transfer and allows time for preimplantation genetic testing).

Although the above refers to studies carried out mainly in IVF with autologous oocytes, when it comes to pregnancies through OD, pooled data report that nearly 40% of them come from FET (23). The former is relevant as it has been argued that the increased risk of preeclampsia found in FET could be linked with the selected protocol for embryo transfer, rather than the cryopreservation and freezing-thawing process itself (74, 75).

Briefly, commonly used protocols for embryo transfer could be summarized in, natural cycles, stimulated cycles, and programmed cycles. In the latter, there is no ovulation associated, therefore no corpus luteum (CL). This becomes relevant as programmed cycles are employed in OD and there is consistent evidence that CL produces not only progesterone and estrogen, but also Relaxin and VEGF. The last two have been found to be implicated in maternal renal and circulatory pregnancy-adaptation and are not replaced during programmed cycles (20, 21, 76). Also, impaired endometrial receptivity has been linked with placental dysfunction among IVF (77). Therefore, it is plausible that the absence of these factors could contribute to an abnormal uterine environment, a suboptimal endometrium support with impaired decidualization, and an insufficient maternal-pregnancy adaptation (78). Thus, leading to the higher risk of placental dysfunction found in pregnancies through OD.

Developmental stage at embryo transfer (i.e., blastocyst-vs. cleavage-stage) has been proposed to influence perinatal outcomes (79, 80). To date, exploring the independent effect of developmental stage at the time of transfer and the impact of cryopreservation on the outcome of interest has been challenging. A recent network meta-analysis (81) demonstrates (with a very-low certainty of evidence) that frozen-blastocyst transfer was associated with a reduction in the risk for LBW compared with both fresh-transfer modalities, and fresh-cleavage transfer may be associated with a reduction in the risk for perinatal death compared with frozen-blastocyst transfer. However, high-quality RCTs and individual participant data meta-analyses are still lacking.

### Preimplantation genetic testing

Similar to the reported increase of pregnancies conceived through ART (1), the use of preimplantation genetic testing (PGT) has demonstrated a progressive increase over time (82, 83). In part due a higher risk of pregnancies with chromosomal abnormalities among patients with advanced maternal age and the possibility of testing for several inherited disorders among patients with recurrent pregnancy loss and recurrent implantation failure, among others (83). Most of PGT are conducted through trophectoderm biopsy, in which 5 to 10 trophectoderm cells are extracted as study samples (83–85). As placenta develops from the trophectoderm (86), there is some concern that the use of PGT could be related to defective placentation and the development of placental dysfunction (87, 88), thus increasing the risk of pregnancy complications such as hypertensive disorder of pregnancy and preeclampsia among others (89, 90). While initial meta-analyses showed that PGT pregnancies were associated with a higher risk of hypertensive disorder of pregnancy, their results were limited by a high sample heterogeneity (91, 92). A most recent systematic review and meta-analysis, restricted only to singletons from FET cycles, including 11.469 live births after PGT and 20.438 live births after IVF/ICSI (no-PGT), concludes that trophectoderm biopsy does not alter the risk of developing hypertensive disorders in subsequent pregnancies (84). Nonetheless, larger cohort studies and well-designed RCTs are still lacking.

Regardless of the above, the use of PGT could be considered at least as non-routine among pregnancies through OD. Since, it has been shown to report no benefit among fresh oocyte donation cycles recipients (93–95), and conflicting results have been reported for frozen oocyte donation cycles recipients (95, 96). Therefore, it seems reasonable not to consider PGT as a major contributing factor for placental dysfunction among OD pregnancies.

### Immune tolerance breakdown

Normal placentation and pregnancy evolution requires the development of maternal immune tolerance to a semi-allogeneic fetus. To date, most accepted mechanisms involved in pregnancy immunomodulation and crosstalk between mother and fetus include; (i) a trophoblast with an overall poor antigenicity, mainly due to a lack of classic HLA-I and II antigens, with the exception of HLA-C, and the expression of nonclassical HLA molecules of class E and G (97, 98); (ii) a shift in the functional balance of T helper (Th) cells towards type-2 cells with a decline in cell-mediated Th1-type immunity (99); (iii) a change in the activity of uterine natural killer (uNK) cells from cytotoxic to regulatory, mainly producing chemokines, growth factors, cytokines and angiogenic factors, of relevance for the development of maternal–fetal interface (100); and (iv) a major proportion of macrophages with an anti-inflammatory, M2-like phenotype, involved in the dampening of immune reactions (98).

Several findings support the role of immunological dysfunction in the development of preeclampsia among spontaneous conception (39, 100). Pregnancy after OD is considered as a unique model to assess the immunologic pathways involved in placental dysfunction, as the fetus is an absolute allograft in contrast to semi-allograft fetus in natural conception.

In line with the above, it has been shown that among OD pregnancies, the degree of HLA mismatch between mother and fetus is correlated with a higher number of maternal decidual-activated CD4^+^ Treg cells (101–104), a reduced number of tissue macrophages (105, 106), and the development of gestational hypertension and preeclampsia (107, 108). Furthermore, the risk of preeclampsia has been reported to be even higher among pregnancies conceived with double gamete donation (oocyte and sperm donation) (109), which could be attributed to an additive effect from the lack of paternal antigen-specific tolerance (97).

Also, genome-wide mRNA analysis in placentas from OD pregnancies have shown a reduced expression of thrombomodulin (110), several complement regulatory proteins (111), and altered immunoregulation by co-inhibitory pathways (112).

Moreover, several placental lesions are observed at different histologic levels in women with pregnancies conceived through OD, supporting an abnormal immune response. Of remark, (i) severe chronic deciduitis with dense fibrinoid deposition is a characteristic finding in OD pregnancy. Suggesting an important maternal alloimmune reaction resembling host versus graft disease at the human fetal–maternal interface (113). (ii) Also, a significantly increased prevalence of VUE is reported among pregnancies conceived through OD (30) which represent a manifestation of maternal anti-fetal rejection. (iii) Of remark, Schonkeren et al. (114) described a specific histologic lesion among uncomplicated OD pregnancies consistent on a diffuse inflammatory infiltrate involving the entire chorionic plate. In their study, preeclampsia occurs only in the group without the immunological lesion. Therefore, this lesion could reflect a protective immune mechanism towards the completely allogeneic fetus.

### Other mechanisms

It is known that there are social determinants for placental insufficiency, being more prevalent among women from disfavoured socioeconomic status (115). The pathways operating these relationships are not fully understood, and epigenetic mechanisms may explain intergenerational transmission (116). A fraction of egg donations is non-altruistically motivated, making donors more likely to come from a more disadvantaged socioeconomic background, which could result in higher rates of perinatal complications in recipients.

## Discussion

The development of ART and specifically the progress achieved in conceiving pregnancies through OD represent a significant opportunity for couples which under other conditions would not be able to achieve pregnancy. However, it should be acknowledged that there is consistent evidence of a higher risk profile among this subgroup of patients.

In this mini review, we intended to succinctly summarize the most widely accepted pathways linked with placental dysfunction. Overall, it could be stated that several non-exclusive physio pathological mechanisms are involved, rendering to these patients a cumulative higher risk of progressive placental dysfunction and preeclampsia.

It is our belief that the subgroup of OD pregnant patients requires further attention. First, already among infertile populations there are reports of higher morbidity & mortality (117, 118). Second, at the population level, there is a progressive and consistent trend of increasing numbers. Theoretically, this could lead to a worldwide higher frequency of preeclampsia. Third, at the individual level, the patient baseline characteristics plus the combination of the physio pathological mechanisms involved could potentially lead to more severe cases (119–121).

Regarding management of ART pregnancies, current recommendation from the Society of Maternal–Fetal Medicine (16) and the UK National Institute of Clinical Excellence (122), consider IVF as a moderate risk factor for preeclampsia and recommends low-dose aspirin and serial scanning only if an additional risk factor is found. However, these guidelines lump together all ART techniques as an overall category, without establishing differences between the mode of conception. Moreover, there are no clear recommendations regarding other surveillance tools, such as maternal and fetal Doppler assessment or angiogenic markers assessment, which arguably have shown moderate-to-good performance for the prediction of adverse perinatal outcomes among high-risk pregnancies (123, 124), and has been proposed as a tool to capture placental dysfunction secondary to pathophysiologic mechanisms other than early defective trophoblast invasion (125, 126).

There are still several research gaps and potential future developments in the field; for one side, there is a need for better characterization and a more complete risk-profile assessment of candidates for OD. In line with the above, identifying novel predictive factors to assess the risk for maternal serious complications may be of value (127). Also, evaluation for signs of immune tolerance breakdown, through the assessment of cellular subpopulations imbalance or its product (such as cytokines or chemokines) (128) and its correlation with known clinical signs of placental dysfunction (i.e., angiogenic markers or fetal & maternal Doppler), could also be explored. Moreover, HLA screening and matching could also be considered as a suitable tool attempting to decrease the reported immune tolerance disbalance (129).

Therapeutic interventions such as the use of some immunosuppressive agents have already shown some encouraging results enhancing outcomes among patients with recurrent pregnancy loss. Among them, hydroxychloroquine is a known anti-inflammatory and immune regulator drug commonly used in patients with autoantibodies disease. Its use during pregnancy has shown to improve the live birth rate in patients with persistent positive antiphospholipid antibodies and to reduce the risk of preeclampsia and fetal loss in mid and late pregnancy among patients with systemic lupus erythematosus (130–132). Also, when combined with prednisone, it has shown to improve outcomes of frozen embryo transfer in antinuclear antibody-positive patients undergoing IVF/ICSI treatment (133). Moreover, its use has been reported as an effective therapeutic strategy in women with repeated implantation failure due cellular immune abnormalities, through a shift in Th2 responses (134). Therefore, hydroxychloroquine could be proposed as a potential treatment for immune tolerance imbalance among pregnancies through OD. However, there is still scarcity of high-quality data that precludes further recommendations (135, 136). Finally, up to date and evidence based counselling about the related short and long-term risk should be offered to OD candidates, as in some cases the risk may be significant, and even overcome the benefits (137–141).

In conclusion, compelling evidence suggests the convergence of various additive factors associated with placental dysfunction in pregnancies conceived through oocyte donation. These factors encompass patient baseline characteristics, absence of corpus luteum, and dysfunction in pregnancy immune tolerance. Further research is imperative as this demographic constitutes a subgroup exhibiting the highest susceptibility to placental dysfunction, potentially necessitating a more vigilant follow-up – a practice not presently endorsed by existing guidelines.

## Author contributions

JC: Writing – original draft, Writing – review & editing. BF: Writing – original draft. FÁ: Writing – original draft. AV: Writing – original draft. MM: Writing – review & editing. FF: Writing – review & editing.
